# 'Learning by doing', a model for improving the promotion of healthy lifestyles by student nurses

**DOI:** 10.1186/s12912-023-01398-3

**Published:** 2023-07-08

**Authors:** Francisco Javier Pérez-Rivas, Milagros Rico-Blázquez, Candelas López-López, Silvia Domínguez-Fernández, José Luis Cobos-Serrano, María Julia Ajejas Bazán

**Affiliations:** 1 Departamento de Enfermería, Facultad de Enfermería, Fisioterapia y Podología, Universidad Complutense de Madrid, Plaza Ramón y Cajal nº3, Ciudad Universitaria, Madrid, 28040 Spain; 2 Grupo de Investigación UCM “Salud Pública-Estilos de Vida, Metodología Enfermera y Cuidados en el entorno comunitario”, Departamento de Enfermería, Facultad de Enfermería, Fisioterapia y Podología, Universidad Complutense de Madrid, Madrid, Spain; 3 Red de Investigación en Cronicidad, Atención Primaria y Promoción de la Salud—RICAPPS—(RICORS), Instituto de la Salud Carlos III, Madrid, Spain; 4 Instituto de Investigación Sanitaria Hospital 12 de Octubre (Imas12), Madrid, Spain; 5 Unidad de Investigación de la Gerencia Asistencial de Atención Primaria, Servicio Madrileño de la Salud, Madrid, Spain; 6 Unidad de Cuidados Intensivos de Trauma y Emergencias, Hospital Universitario 12 de Octubre, Madrid, Spain; 7 Madrid Salud, Ayuntamiento de Madrid, Madrid, Spain; 8 Consejo General de Enfermería, Madrid, Spain; 9 Academia Central de la Defensa, Escuela Militar de Sanidad, Ministerio de Defensa, Madrid, Spain

**Keywords:** Learning, Lifestyle, Nursing competencies, Nursing Education Research, Nursing Process, Quasi-experimental study, Students, Nursing

## Abstract

**Background:**

'Learning by doing' is a learning model based on performing actions and gaining experience. The 'nursing process' is a systematic, rational method for providing nursing care. During their university education, nursing students need to acquire the ability to promote healthy lifestyles.

**Objective:**

To determine the effectiveness of a learning strategy based on learning by doing and grounded in the use of the nursing process, on the lifestyle of nursing students.

**Methods:**

This quasi-experimental intervention (before-after), performed over 2011–2022, involved 2300 nursing students at a university nursing school in Spain. The risk factors for chronic diseases—being a smoker, being overweight, or having high blood pressure—to which each student was exposed were recorded. Those positive for at least one risk factor selected companion students as 'support nursing students' who became responsible for designing an individualised care plan to reduce the risk(s) faced. To ensure the correct use of the nursing process, teachers approved and monitored the implementation of the care plans. Whether risk-reduction objectives were met was determined three months later.

**Results:**

The students with risk factors largely improved their lifestyles (targets for reducing smoking/body weight were met) with the help of their supporting peers.

**Conclusions:**

The learning by doing method demonstrated its effectiveness, improving the lifestyle of at-risk students via the use of the nursing process.

## Introduction

### Learning by doing

Based on the postulates of the American philosopher and educator John Dewey, ‘learning by doing’ is a model that holds learning to arise from an individual's experimentation in finding solutions to a problem and the consequences of acting in a certain way [1]. This model is different to traditional teaching. Rather than memorizing information and remaining largely a spectator of knowledge transfer, the students themselves become agents that build and acquire knowledge [2]. ‘Learning by doing’ is based on action, experience, and teamwork, and grounded in the idea that people learn from what they do, that theory reinforces what they learn, and that the entire learning process is more effective and enjoyable when undertaken as part of a group. Students therefore acquire an active role in learning by doing. This learning model also incorporates innovative educational techniques (project- or problem-solving-based learning, cooperative work, etc.) and encourages self-assessment. The teacher, meanwhile, plays the role of moderator and generator of adequate environments in which learning can take place [3].

Terms such as 'learning by doing', 'project- and/or problem-based learning' or 'situated learning' are variations on the same theme. All are based on the idea of active learning, centered on the learner's practical activity and experience [4].

Numerous attempts have been made in different countries to implement and develop active learning models [5–8], particularly learning by doing [9–14].

### The nursing process

Care is the essence of nursing. The care delivered by nurses should aim to promote health, prevent illness, relieve pain, and aid rehabilitation, via actions based on interpersonal relationships and the use of specialised scientific and technical knowledge [15]. The nursing process is the model that describes how professional care is given, based on the available evidence, by nurses to individual patients, families and communities. It is a systematic, humanistic, and rational means of competent care delivery [16]. A nursing care plan is the record of the nursing process; along with other uses, it serves as a communication tool for ensuring the continuity of care.

### Lifestyle and disease burden

Lifestyle is a major determinant of health. It includes all habits and behaviours that affect daily life. When a person's lifestyle is inadequate or unhealthy it increases the risk of developing chronic disease and has important costs in terms of morbidity, mortality and disability, as well as economic consequences. Indeed, chronic disease cause an overall 74% of all deaths, rising to 92.8% in Spain [17]. Most such disease is associated with modifiable risk factors, such as smoking, a high body mass index (BMI), poor diet, and a sedentary lifestyle [18]. Several recent studies have reported that, among the general population, those with a healthy lifestyle are at lower risk of all-cause mortality [19] and have a longer life expectancy [20]. Adolescents and young people with healthy lifestyles are more likely to reach adulthood with favourable markers for cardiovascular disease [21].

### Competencies of nursing graduates

The European Higher Education Area has the aim of bringing university teaching closer to professional practice via the implantation of a learning model based on capacitation, in which the student acquires necessary knowledge but also develops the ability to perform well in the work environment. Order CIN/2134/2008 of the Spanish Ministry of Science and Innovation counts the following among the capacities of nursing graduates: to promote healthy lifestyles and self-care by encouraging the maintenance of preventive and therapeutic behaviours, and to apply the nursing process to guarantee well-being and provide safe, quality care to patients.

In our faculty, students receive training in the use of the nursing process and nursing taxonomies in the subject 'Methodology of Nursing Practice', which is taught in the second semester of the first year. Students must develop the competence of 'Using the nursing process as a method of decision-making for the solution of health problems of the person, family and community' and of 'Planning care appropriate to the needs of the patient, evaluating the achievements obtained', all this by means of the appropriate use of different nursing taxonomies.

### The 'Look after yourself, look after others' seminar

Our faculty's Community Nursing course forms part of the study plan for a first degree in Nursing. It offers six credits within the European Credit Transfer and Accumulation System (ECTS) and is taught in the second semester of year II. One of the major objectives of the course is that students understand the most common chronic health problems and the main means via which they may be prevented, to be able to detect them at an early stage, and to prevent the appearance of complications. The capacities developed in this course include the promotion of a healthy lifestyle through the use of the nursing process. To help acquire these capacities, the course includes a starting seminar entitled 'Take care of yourself/Take care of others'. Its aim is to sensitize students to the importance of risk factors in the development of chronic disease and the role of nurses in their control. Following an active participation methodology, students can bring theory and practice together, modify their attitudes and develop new lifestyle-changing skills. The seminar is held over the first two weeks of term. Attendance is obligatory and lasts 3 h, during which time the risk factors related to chronic diseases shown by the students are identified. This requires their teachers to determine the students' height, weight, blood pressure and smoking habit.

The aim of the present work was—as part of the above course unit—to determine the effectiveness of a learning strategy based on learning by doing and grounded in the nursing process, on the improvement of lifestyle (reduction in risk factors for chronic disease) in students of nursing.

## Methods

### Design and study period

This quasi-experimental intervention was performed over an 11-year period (2011–2022) at a university nursing school in Spain. The study was conducted using neither randomization nor a control group. Repeated measures were carried out before and 3 months after the intervention to check for the achievement of objectives.

### Subjects and recruitment

The study subjects were university students enrolled on the Community Nursing course (intake years 2011/2012 until 2021/2022) who attended the Take care of yourself/Take care of others seminar. Students repeating the course or who failed to attend the seminar were not included in analyses. Students who were pregnant were also excluded since their BMI and blood pressure, etc. may have been altered by gestation.

### Study variables

The recorded variables included student age and sex, body weight and BMI, systolic and diastolic blood pressure (SBP and DBP, respectively), number of cigarettes smoked per day, and, in smokers, the carbon monoxide (CO) in exhaled air (determined via cooximetry). The risk factors recorded were overweight (BMI ≥ 25 to < 30), obesity (BMI ≥ 30), high blood pressure (≥ 140/90 mmHg) and smoking (i.e., smokers with an exhaled air CO concentration of ≥ 10 ppm).

### The intervention (Fig. 1)

**Fig. 1:**
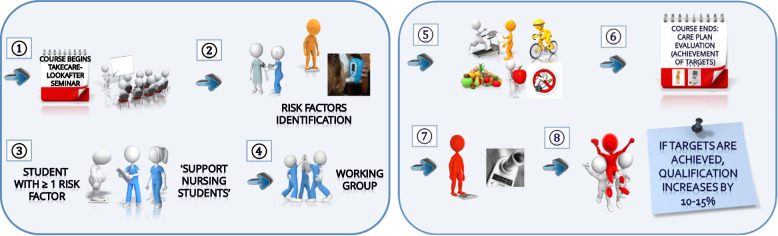
Study plan

Students who were overweight/obese or who were smokers were encouraged to modify their risk factors and acquire a healthier lifestyle. In response, interested students voluntarily chose one or two classmates to act as supporting student nurses (SSNs), who then became responsible for designing their patient student's risk-factor-reducing care plan. All students in the groups thus formed had periodic meetings over a three-month intervention period to monitor progress.

To ensure the correct use of the nursing process, all the care plans made by the students had to meet the approval of the subject teachers, who are specialist nurses in family and community nursing with extensive experience in the use of the nursing process [15, 22, 23]. Throughout the process, the lecturers tutored the work to ensure the proper implementation of the care plan. All care plans were based on the use of nursing taxonomies (NANDA for nursing diagnoses, NOC for outcome criteria, and NIC for interventions).

At the end of the intervention period, the students presented the programmes they had implemented, in coordination with the teachers of the subject, and assessed the achievement of their objectives (measuring the body weight of the student patient and re-performing the cooximetry test for the smokers). The intervention was considered positive if: 1) Smokers quit their habit or reduced their cigarette consumption to achieve an exhaled air CO concentration of < 6 ppm (a normal value for non or sporadic smokers); or 2) overweight/obese students lost 3 kg over the three months intervention period (I kg per month; international recommendations consider a loss of 1–1.5 kg per week to be safe; those who lose weight slowly also do better at not regaining it [24]). Reduction in blood pressure was not considered since this would have required confirmatory diagnosis and monitoring at a health centre. Indeed, those with high blood pressure were referred to their local health centre for follow-up.

By way of incentive, students who achieved the objectives set (reduction in smoking/weight loss) were awarded a 15% higher grade for this course unit, while successful SSNs were awarded an extra 10%. The students who did not take part (either because they had no risk factors, were excluded as described above, or were not chosen as SSNs) performed work involving the determination of cardiovascular risk and the planning of appropriate care, allowing them a 10% grade increase.

### Statistical analysis

A descriptive analysis was made of the student patients' sociodemographic and anthropometric variables, and risk factors. Means and standard deviations, plus 95% confidence intervals (95%CI) were determined for quantitative variables. Qualitative variables were described as absolute and relative frequencies. The Student t-test for paired samples was used to examine the differences in quantitative variables before and after the intervention (weight and exhaled air CO concentration). All calculations were made using SPSS Statistics v.25.0 software.

### Ethical considerations

The study was approved by the Research Committee of our faculty and by the Ethics in Research Committee of a University Hospital in Madrid, Spain (Decision Number: 21/408_R). All nursing students who participated were informed of the nature of the study by the researchers/teachers. The aim of the study and the data collection procedures were explained before the start of the seminar. Verbal informed consent was obtained from each student. All work was performed in accordance with the principles of the Declaration of Helsinki (2013 version). All data were treated adhering to the General Data Protection Regulation (GDPR) 2016/679, 27th April 2016, and the Spanish *Ley orgánica de protección de datos y garantía de derechos digitales* (LOPDGDD; Data Protection And Guarantee Of Digital Rights) 3/2018, 5th December.

## Results

Of the 2300 students enrolled in the Community Nursing course between 2011 and 2022, 113 were excluded (73 repeating students, 3 pregnant students, and 37 who did not attend the required seminar). The 2187 remaining students—82.1% of whom were female—all took part. Their age was 22.5 ± 6.7 years.

### Risk factors

A total of 949 students (43.4%) had at least one risk factor: 432 (19.8%) were smokers (consumption 7.0 ± 5.3 cigarettes per day), 517 (23.6%) had excess body weight (409 [18.7%] were overweight, and 108 [4.9%] were obese [BMI 23.3 ± 3.8, range 15.3–44.4]), and 145 had a blood pressure higher than ≥ 140/90 mmHg (these students were referred to their local health centres for monitoring). Thirty-six students with a BMI of < 17.5 were also referred for monitoring. A total of 193 students (8.8%) had more than one risk factor.

### Formation of work groups (Fig. 2)

**Fig. 2:**
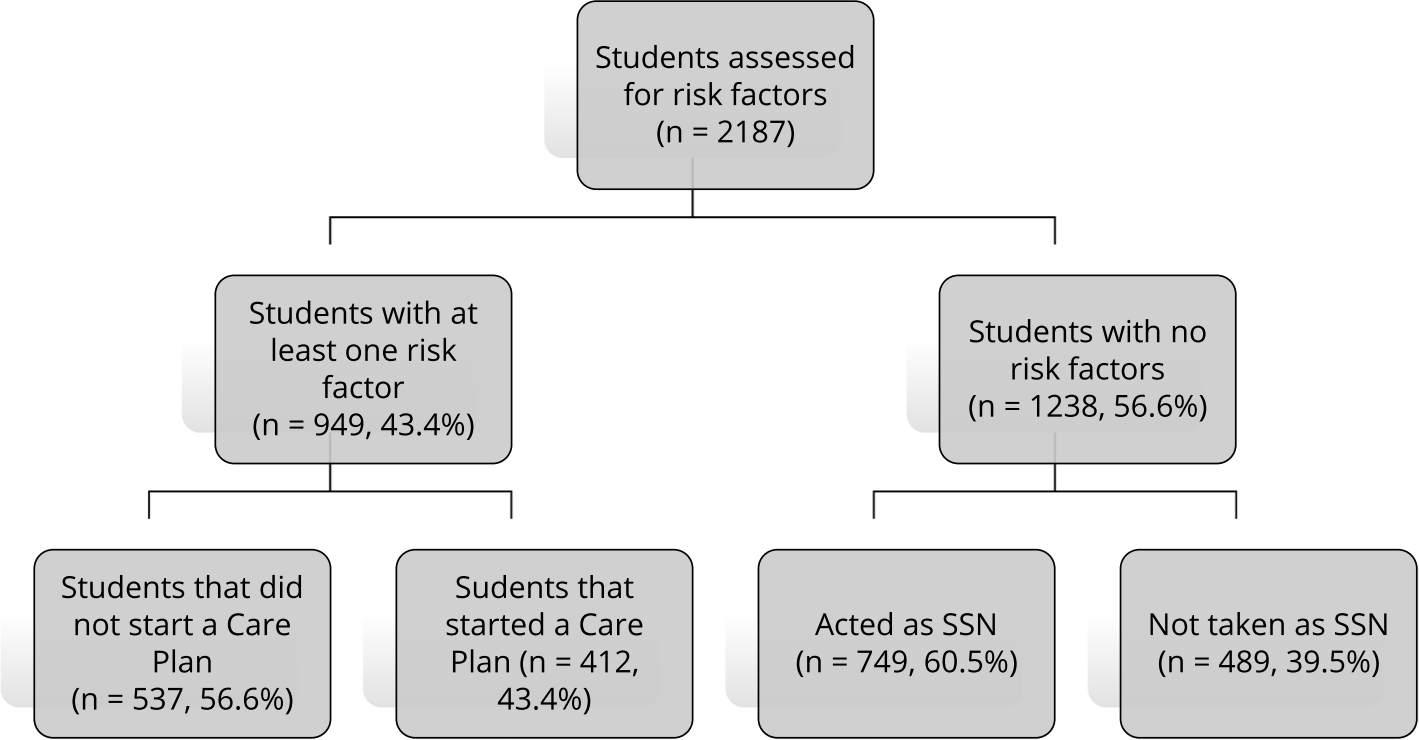
Study flow-chart

Of the 949 students with at least one risk factor, 412 (43.4%) voluntarily decided to start a program designed to modify their lifestyle; 114 decided to try to quit smoking (26.4% of the total number of smokers), and 298 of those who were overweight/obese (57.6% of the respective total) decided to try to lose weight. These 412 students were able to count on 749 companions who would act as SSNs for three months. The total number of students involved in the intervention in one form or another was therefore 1161 (53.1% of all who attended the seminar).

### Effectiveness of the intervention (Fig. 3)

**Fig. 3:**
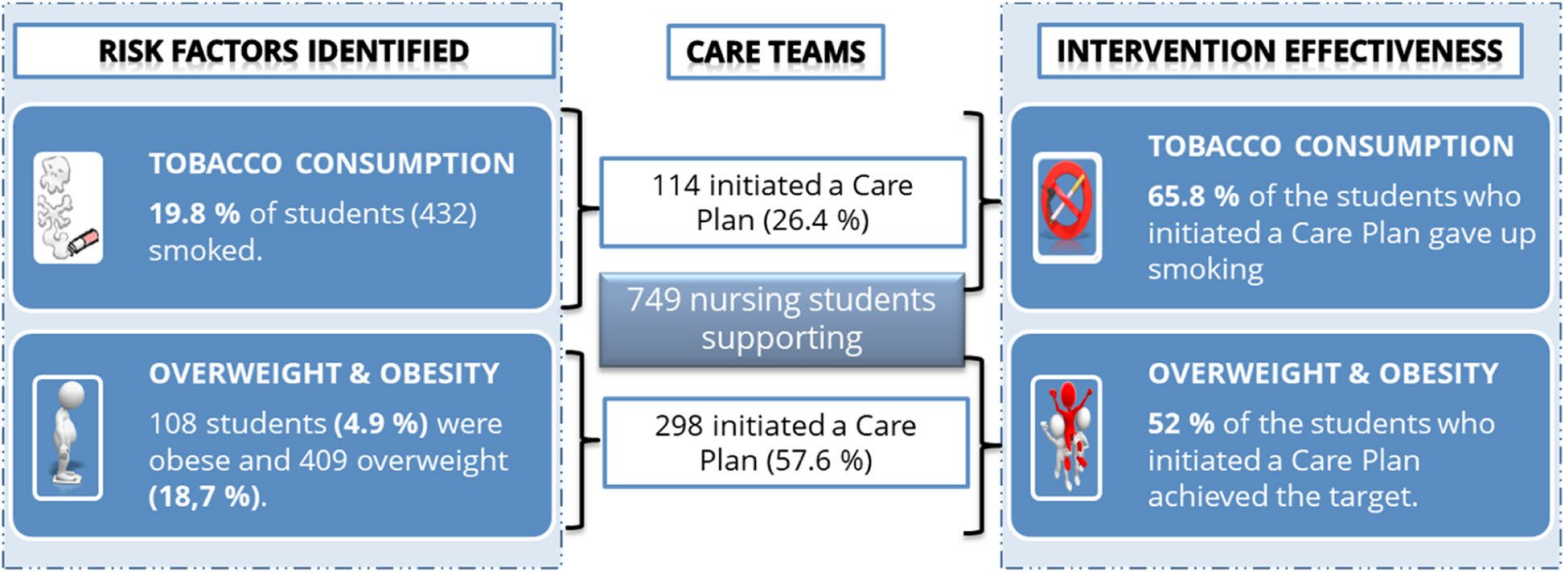
Study outcomes for reduction of tobacco consumption and overweight/obesity

Of the 412 students who started a care plan, 230 (55.8%) reached the objectives set for them. The intervention was most effective in reducing smoking; 65.8% (75 students) either stopped smoking or became only occasional smokers (cooximetry < 6 ppm). The difference in the pre- and post-intervention mean cooximetry values was 10.6 ppm (95%CI 8.7–12.6; *p* < 0.001). A total of 155 (52%) who tried to lose weight achieved the set objective of losing 3 kg. The mean weight loss was 4.8 kg (95%CI 4.6–5.1; *p* < 0.001).

## Discussion

The prevalence of risk factors for chronic disease among the students was high, similar to results reported for other university students [25, 26]. It should be noted that most university students are in the last stage of adolescence or in the 'young adult' stage. During this time of life, behaviours followed during infancy become consolidated, and others are incorporated within new socialization contexts [27]. Habits acquired during this time, however, are usually maintained throughout adulthood, and changing them later becomes difficult [28]. It is therefore vital that health be promoted during this time of life if risk factors are to be eliminated and a healthy lifestyle adopted.

It should be remembered that the present subjects were student nurses, and in a few years would be health professionals, making them reference points for the people with whom they will interact. They will become responsible for recommending their patients follow a healthy lifestyle. There is evidence to suggest that the lifestyles followed by health professionals have a vital influence when advising patients to make lifestyle changes [29]. Health professionals who look after themselves better have a better chance of instilling the same behaviour in their patients, while those who do not seem to have less capacity to achieve this [30]. These findings invest the present results with special importance,in this work the lifestyles of many student nurses were improved, perhaps making them better able to achieve the same in their future patients.

The students who acted as SSNs were particularly involved in their role to have their student patients modify their risk factors; not only did they gain personal satisfaction in seeing their patients' health improve, but they could also improve their grade. The fact that they had to design a care plan based on the nursing process added value to their experience since this is what practising nurses would have to do, and there is evidence to show that such training helps them become competent [15, 31].

The literature contains no information on the use of active learning for modifying the lifestyle of university students, but several studies report the effectiveness of such strategies at this level regarding aspects related to student performance [7, 32] including interest, the capacity to learn [5], attitude [6], involvement, satisfaction [8], the transfer of theoretical knowledge, critical and reflective thinking [33], skills learned and dedication to learning [3]. Several studies also report advantages to active learning that involve experimentation and personal involvement in the training of nurses. For example, Hill [34] reported a teaching technique based on learning by experiment to be very effective in students' acquisition of clinical skills and performance. Similarly, Shin et al. [35], who examined the development of different types of capacity by nursing students depending on learning style, found that those exposed to active learning returned better results than those exposed to traditional teaching methods. This was particularly true with respect to the acquisition of clinical skills and critical thinking.

This style of learning not only returns good results in terms of academic performance and the acquisition of skills and capacities, but students report greater satisfaction [36]. However, despite all this evidence, passive teaching methods continue to dominate nursing education [37]. Strategies need to be developed to allow active learning to be implanted more widely.

A major limitation of the present work is the lack of a control group to check how risk factors may have been altered in those not involved in the intervention. Neither can the study's short-term follow-up determine whether any changes in lifestyle are maintained over the long-term.

## Conclusions

Universities have a role to play as promoters of health. Alongside other authorities, they should help in the establishment of healthy lifestyles, and thus contribute to the personal wellbeing—as well as academic and professional development—of their students. The present learning by doing strategy helped to improve the lifestyle of nursing students with risk factors for chronic diseases by using the nursing process. This experience can be useful for all students, but especially for those working in the community setting. This type of learning could be advantageous in many areas of nursing education.

## Data Availability

The datasets produced during the current study are available from the corresponding author upon reasonable request.
